# Cancer-Associated Fibroblasts’ Functional Heterogeneity in Pancreatic Ductal Adenocarcinoma

**DOI:** 10.3390/cancers11030290

**Published:** 2019-03-01

**Authors:** Mohammad Awaji, Rakesh K. Singh

**Affiliations:** 1 Department of Pathology and Microbiology, University of Nebraska Medical Center, 985845 UNMC, Omaha, NE 68198-5845, USA; Mohammad.awaji@unmc.edu; 2 Department of Pathology and Laboratory Medicine, King Fahad Specialist Hospital-Dammam, Dammam 31444, Saudi Arabia

**Keywords:** pancreatic cancer, PDAC, cancer-associated fibroblast, myofibroblast, inflammation, IL-6, CXCL8, TGF-β

## Abstract

Pancreatic ductal adenocarcinoma (PDAC) is a leading cause of cancer-related deaths in the USA. Desmoplasia and inflammation are two major hallmarks of PDAC. Desmoplasia, composed of extracellular matrix (ECM), cancer-associated fibroblasts (CAFs), and infiltrating immune and endothelial cells, acts as a biophysical barrier to hinder chemotherapy and actively contributes to tumor progression and metastasis. CAFs represent a multifunctional subset of PDAC microenvironment and contribute to tumor initiation and progression through ECM deposition and remodeling, as well as the secretion of paracrine factors. Attempts to resolve desmoplasia by targeting CAFs can render an adverse outcome, which is likely due to CAFs heterogeneity. Recent reports describe subsets of CAFs that assume more secretory functions, in addition to the typical myofibroblast phenotype. Here, we review the literature and describe the relationship between CAFs and inflammation and the role of the secretory-CAFs in PDAC.

## 1. Introduction

Pancreatic ductal adenocarcinoma (PDAC) is one of the leading causes of cancer-related deaths in the United States. The late diagnosis, often after the disease has disseminated, and the limited efficacy of the chemotherapy for advanced disease, are the major challenges in PDAC. Moreover, resistance to therapy and recurrence are frequent, even for patients diagnosed with localized tumors [1]. PDAC is highlighted with a dense and firm desmoplasia, composed of extracellular matrix (ECM) deposition and infiltrating leukocytes, endothelial cells, and cancer-associated fibroblasts (CAFs). Desmoplasia is implicated in PDAC development, progression, and dissemination, as well as therapy resistance [2,3,4]. Resolving desmoplasia has been attempted through digesting ECM, targeting CAFs, or inhibiting desmoplasia-associated pathways [5,6,7,8]. Some of these attempts produced accelerated tumor progression and worsened prognosis [6,7], which implies that there is more to desmoplasia than we currently know.

CAFs are the major contributor to desmoplasia, producing ECM and multiple soluble factors that contribute to tumor progression [2,9,10]. Although CAFs are often treated as a single entity, they are vastly heterogeneous by origin. There is an agreement that CAFs have a mesodermal origin, but their molecular definition is still debatable. Currently, CAFs represent cells present in the tumor microenvironment that are not tumor cells, leukocytes, endothelial cells, or epithelial cells and that carry fibroblastic features such as the expression of fibroblast-specific protein 1 (FSP-1) [11].

In PDAC, pancreatic stellate cells (PSCs) are the most studied CAFs subtype. Stellate cells, referring to their star-like shape, are found in several organs, including the kidneys, lungs, intestines, spleen, uterus, and skin, but they are mainly described in the liver and pancreas [2,10,11,12]. PSCs are found in the periacinar, perivascular, or periductal regions of the exocrine pancreas. In normal conditions, PSCs are usually in the quiescent state, with long cytoplasmic extensions and vitamin-A storing fat droplets. PSCs express many markers, including the intermediate filament proteins desmin and Glial fibrillary acidic protein (GFAP) that, along with vitamin-A storing droplets, can distinguish them from normal fibroblasts [10,12]. PSC markers also characterize several other cell types such as desmin that is seen in monocytes, GFAP of astrocytes, vimentin that also characterizes leukocytes and endothelial cells, and nestin of neuroepithelial stem cells [10]. Activation of PSCs occurs as a result of milieu changes such as pancreatic injury, or in response to secreted factors such as platelet-derived growth factor (PDGF) and transforming growth factor beta (TGF-β) [2,10,12]. When activated, PSCs assume the myofibroblast-like phenotype by upregulating α-smooth muscle actin (αSMA) and collagen I and losing their vitamin A-storing fat droplets, in addition to increased nucleus size, prominent ECM production, and increased cell proliferation and migration potentials [2,10,12,13]. Additional reports indicated that activated PSCs express fibroblast-activation protein α (FAP) [2,12,14]. Activated PSCs play important roles in pancreatic repair following injury and acute inflammation via modulating ECM production and tissue remodeling [10,12]. Following the cessation of pancreatic assault, activated PSCs revert into quiescence or undergo apoptosis. Repeated assaults and chronic pancreatic inflammation cause sustained PSCs activation, which increases the risk of fibrosis and cancer [10,15].

Tissue-resident fibroblasts can also contribute to CAFs population [11]. A subset of normal fibroblasts found to express the glycoprotein Thy-1 were able to differentiate into CAFs after treatment with TGF-β. Genetic mutations, such as the inactivation of the tumor protein 53 (p53) and the phosphatase and tensin homolog (PTEN), have been frequently observed in stromal cells and can also turn them into CAFs [16]. Moreover, CAFs can arise by transdifferentiating through epithelial-mesenchymal transition (EMT) or endothelial to mesenchymal transition [11,16], but more direct sources of CAFs include bone marrow-derived fibrocytes, mesenchymal stem cells, and adipocytes [11,16]. These diverse origins of CAFs can explain the absence of consensus on a molecular definition. Nonetheless, multiple markers have been widely used to distinguish CAFs, including PDGF-receptor-β (PDGFR-β), alpha smooth muscle actin (αSMA), and FAP [2,12,14]. These markers are not uniformly expressed in all CAFs [11], which can be due to the presence of CAFs concurrently at multiple differentiation stages or because of the diverse origins of CAFs. The coexistence of multiple subsets of CAFs could explain the diverse roles and abilities they carry out to promote tumorigenesis and progression and could explain why targeting CAFs using a single marker, such as αSMA, can have an adverse outcome [6]. CAFs have been described to be versatile and to have a wide range of roles in cancer [10]. It is not clear, however, if all the roles can be carried out by all CAFs, or if the versatility is due to CAFs diversity. A better understanding of different CAFs subsets could greatly impact our ability to target desmoplasia safely. In this review, we discuss the functional heterogeneity of CAFs and how the abundance of certain subsets can impact tumor progression or lack thereof.

## 2. Role of CAFs in PDAC

PDAC develops as a result of a progressive accumulation of genetic alterations in multiple oncogenes and tumor suppressor genes. Oncogenic Kristen rat sarcoma viral oncogene homolog (oncogenic Kras) occurs very early, preceding PDAC precursors. The late events of inactivating tumor suppressors such as p53 and the Mothers against decapentaplegic homolog 4 (Smad4) allow progression to invasive PDAC [17,18,19]. Although mutations are essential for the malignancy, they do not render them autonomous. Numerous survival, growth, and invasion cues are obtained through cellular and molecular interactions with other components in the tumor microenvironment. CAFs have been implicated in multiple hallmarks of cancer including sustained proliferative signaling, tumor-promoting inflammation, and invasion and metastasis [2,12,20]. In some cancers, the accumulation of CAFs and ECM changes were observed prior to tumor formation, which indicates that CAFs recruitment is essential for tumor development and may be a prerequisite [21,22,23]. The most notable adverse contribution of CAFs to the tumor is acting both physically and biochemically to hinder drug delivery and impose resistance. CAFs produce ECM molecules such as collagen, fibronectin, and hyaluronan [14,24]. The increased deposition of such molecules physically impairs drug delivery to the tumor [25,26]. Inhibiting the Hedgehog (HH) pathway, a major promotor of desmoplasia, and using enzymatic digestion of desmoplasia facilitates drug delivery and increases the intratumoral concentration of the chemotherapy agent [5,8]. Moreover, factors secreted by CAFs such as hepatocyte growth factor (HGF), interleukin (IL)-6 and C-X-C motif ligand (CXCL)8 have been implicated in therapy resistance either by activating resistance-associated pathways or inducing stemness in tumor cells [10,27,28,29,30].

CAFs are also tied to PDAC metastasis. Increased tumor stiffness as a result of increased ECM depositions can increase tumor cells’ contractility, thus allowing tumor cell detachment and invasion [31]. Biochemically, CAFs can play a role in up-regulating EMT that endows tumor cells with more migratory and invasion potentials. Tumor cells co-cultured with CAFs had a fibroblast-like appearance, increased migration, and expressed mesenchymal markers Vimentin, Snail-1, and Zeb [32]. One proposed mechanism for CAFs-induced EMT involves TGF-β that is highly produced by myofibroblasts [33,34].

CAFs involvement in PDAC also extends to tumor growth, proliferation, and nourishment, as well as immunosuppression and immune evasion [2,12,35]. But, is targeting desmoplasia or CAFs a solution for resolving PDAC aggressiveness? There are conflicting reports on the usefulness of targeting CAFs. Olive et al. observed increased vascularization and improved drug delivery, as well as decreased αSMA cells and improved the overall survival of the test mice in response to an inhibitor that targets the HH pathway [5]. The drug, however, when put into the test in a clinical trial, rendered a decreased survival [36,37]. Ozdemir et al. developed a mice model that was depleted of αSMA cells. This model demonstrated an accelerated PDAC with reduced survival, undifferentiated tumors, increased chemotherapy resistance, stemness, and immunosuppression [6]. Rhim et al. targeted desmoplasia by inhibiting the HH pathway. In this model, PDAC exhibited tumors with undifferentiated histology, increased vascularity and proliferation, and reduced survival and myofibroblast infiltration [7]. Together, these independent experiments demonstrate that inhibiting myofibroblasts results in aggressive PDAC with intense immunosuppression, heightened proliferation, tumor stemness, and therapy resistance.

We have discussed the heterogeneity of CAFs based on their origin, however, it is not clear if they present with functional diversity within the tumor and if their origin impacts their function. Ohlund et al. described a distinct subset of CAFs in PDAC with a secretory function that is different from the typical myofibroblast CAFs [38]. These newly described CAFs are characterized with increased secretion of inflammatory mediators, particularly IL-6, and decreased expression of αSMA, in addition to their ability to promote tumor cell proliferation [38]. Thus, CAFs heterogeneity in PDAC can explain why particularly targeting myofibroblasts can render a more adverse outcome. It is not clear at this point if other functional subsets, other than myofibroblast and secretory CAFs, present. In the following sections, we will discuss the contexts by which myofibroblasts or secretory CAFs develop and their impact on the tumor outcome.

## 3. Myofibroblast CAFs

### 3.1. Overview

For a long time, CAFs and myofibroblasts were considered synonymous in the context of cancer and often used interchangeably. We know now that is not accurate. Besides cancer, myofibroblasts are often described in the context of wound healing, in which quiescent fibrotic cells get activated to undertake tissue repair and remodeling. Cancers are often referred to as “wounds that do not heal” [39]. Looking into the wound healing process can provide insights into the dynamics of CAFs activity in cancer.

### 3.2. Wound Healing

Tissue injury causes plasma leakage from local blood vessels. Shortly after, extravasated plasma initiates wound sealing by forming a clot of fibrin, fibronectin, and platelets to trap the blood inside. The sealant clot acts as a provisional scaffold for the migration of inflammatory cells recruited through factors secreted from the damaged tissue cells as well as the platelets. Inflammatory cells clear debris and infectious agents and degrade the clot. Next, activated fibroblasts form granulation tissue by depositing ECM molecules such as collagen, glycosaminoglycans, and fibronectin. Fibroblasts also enable vascularization by recruiting and modulating endothelial cells. Finally, before they disappear, fibroblasts remodel the granulation tissue, allowing a few blood vessels and dispersed fibrocytes in the dense collagenous scar that replaced the collapsed tissue [39,40]. As it appears, wound healing is a very coordinated process. First platelets modulate provisionally sealing the wound and recruiting inflammatory cells. Next, neutrophils, then macrophages, clean the mess before allowing fibroblasts to generate the permanent sealant. Cytokines and chemokines coordinate the timely recruitment and activation of different cells.

Interestingly, ECM deposition and remodeling by fibroblasts occurs after the cessation of inflammation [40]. Inflammation during wound healing, in particular neutrophils and macrophages, happens in two phases. Neutrophils are among the first responders, recruited mainly through C-X-C motif chemokine receptor (CXCR)1/2, to clear the infectious aggressors [41,42]. Classically-activated macrophages (M1) are known pro-inflammatory cells that ingest and degrade tissue debris, pathogens, retired neutrophils, and ECM scaffold to set the stage for tissue repair. Alternatively-activated macrophages (M2; the pro-repair and the anti-inflammatory counterparts of M1) produce cytokines that dampen the inflammation, including IL-10 and TGF-β [43,44,45]. The latter is known to activate myofibroblasts and induce ECM deposition and remodeling [2,10,12,40], the last step in tissue repair. Fibroblasts in wound healing are mainly described as myofibroblasts that are responsible for ECM deposition and remodeling, but it is not clear if other subsets of fibroblasts present with distinct roles similar to those found in cancer that amplify inflammation.

### 3.3. The Context of Myofibroblasts in PDAC

Several secreted mediators, such as PDGF and TGF-β, are considered to have ties to the development of myofibroblasts from quiescent fibrotic cells [2,10,12]. TGF-β typically signals through the Smad pathway. Smad4, also known as deleted in pancreatic cancer 4 (DPC4), is commonly inactivated in PDAC [46]. TGF-β, a multifunctional cytokine, is often found in in the extracellular matrix and is produced by macrophages, lymphocytes, fibroblasts, epithelial cells, and platelets [47,48]. TGF-β is important in prenatal and postnatal development, organ maintenance and homeostasis, and wound healing [47,48]. Intact TGF-β/Smad4 signaling works as a tumor suppressor by blocking cell cycle progression, inducing apoptosis of epithelial cells, and maintaining genomic integrity and tissue hemostasis [47,48,49]. Smad4 inactivation results in ligand accumulation that signal in tumor cells (in a Smad-independent manner) as well as in stromal cells. Non-Smad TGF-β pathways mediate EMT, cytoskeletal organization, and motility through pathways including Extracellular signal-regulated kinases(Erk)/Mitogen-activated protein kinases (MAPK) and other pathways [46,49]. In fibroblasts, TGF-β is known to induce activation and ECM deposition. Furthermore, sustained TGF-β inhibits the synthesis of matrix metalloproteinases (MMPs), thus inhibiting degradation of the newly synthesized ECM. In PDAC, elevated TGF-β levels are found in both plasma and tumor tissues. Overall, TGF-β regulates EMT and tumor stiffness and correlates with metastasis and poor survival in PDAC [46,49,50,51,52,53,54,55,56].

Another molecule that has been linked to myofibroblasts is PDGF. Many reports tie PDGF to fibroblast activation and ECM synthesis along with TGF-β, however, the effect of PDGF is not the same as TGF-β [10,12]. Besides enhancing the proliferation of activated fibroblasts, PDGF plays a significant role in blood vessel formation and maintenance [57,58]. PDGF is mainly secreted by activated platelets but can also be produced by other cells such as macrophages and endothelial cells [57,58,59].

**Figure 1:**
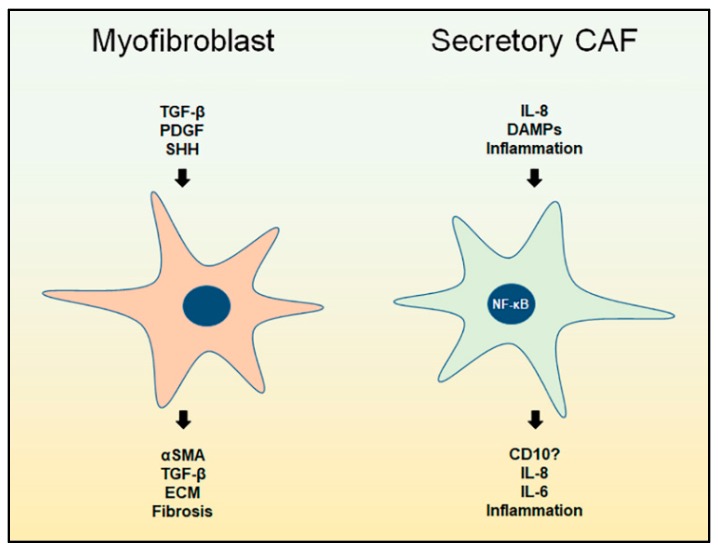
Cancer-associated fibroblasts (CAFs) functional subsets in pancreatic ductal adenocarcinoma (PDAC). Myofibroblasts promote fibrogenic conditions, including increased extracellular matrix (ECM) production, and develop in response to several stimuli, including transforming growth factor beta (TGF-β), platelet-derived growth factor (PDGF) and sonic hedgehog (SHH). Secretory CAFs contribute to inflammation and develop in response to inflammatory mediators, including C-X-C motif ligand (CXCL)8 and damage-associated molecular patterns (DAMPs).

HH molecules, including sonic (SHH), Indian and desert HH, are morphogens that play a crucial role in embryologic growth and tissue morphogenesis. SHH is implicated in wound healing and repair [60,61]. In cancer, SHH is highly implicated in desmoplasia and disrupting the HH pathway was shown to reduce myofibroblasts (αSMA^+^ cells), reduce ECM deposition, and enhance angiogenesis and drug delivery [5,7,62,63,64,65]. Several other molecules have been linked to fibroblast activation, however, there is not enough evidence to link them to a certain CAFs subset (Figure 1).

**Figure 2:**
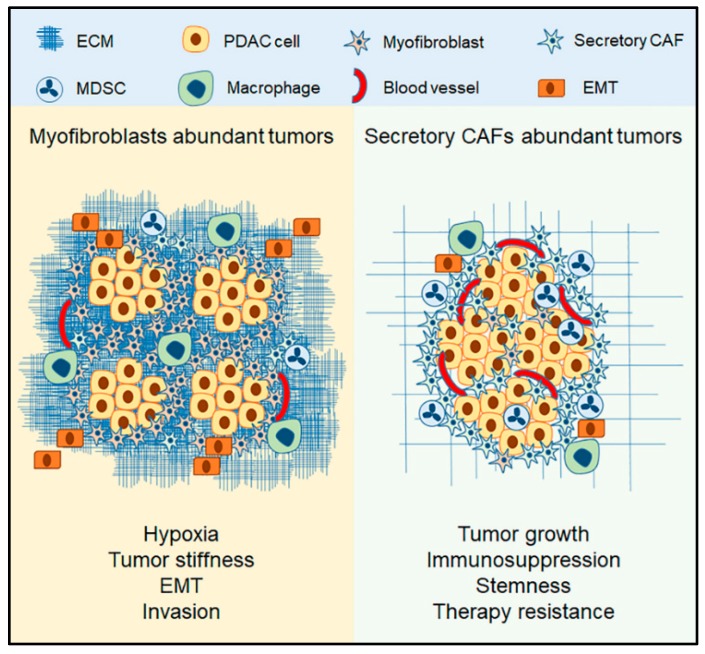
CAFs distribution affects the tumor outcome. Tumors abundant in myofibroblasts are characterized by increased ECM deposition that contributes to hypoxia and tumor stiffness, increased epithelial-mesenchymal transition (EMT), and tendency for metastasis. Abundance of secretory CAFs contributes to tumor growth as a result of vascularization and proliferation cues, promotes immunosuppression by recruiting myeloid-derived suppressor cells (MDSCs), and enhances chemotherapy resistance by increasing stemness.

In summary, several molecules, including TGF-β, PDGF, and SHH, cooperate to establish and maintain desmoplasia by promoting myofibroblasts-phenotype in CAFs. The abundance of myofibroblasts is associated with ECM synthesis and deposition, tumor stiffness, EMT augmentation, and invasion and metastasis (Figure 2).

## 4. Secretory CAFs

### 4.1. Overview

Ohlund et al. identified the presence of two distinct phenotypes of CAFs in PDAC. The typical myofibroblasts (αSMA high) with high ECM synthesis were found adjacent to the tumor cells. The other phenotype that they referred to as inflammatory CAFs (αSMA low) were found at a distance from tumor cells and had a lower ECM expression and a higher expression of inflammatory mediators, particularly IL-6. Inflammatory CAFs possessed the ability to induce tumor cell proliferation [38].

It was established that CAFs secrete several paracrine factors to modulate both inflammatory and fibrotic processes [2,10]. This, however, was attributed to the plasticity and versatility of CAFs and to their ability to carry out multiple roles at the same time. The notion of specialized CAFs subsets is fairly recent and largely understudied. Nonetheless, several reports have pointed, without directly concluding, toward the ability of CAFs to be secretory in certain contexts. We will first discuss the relationship between inflammation and CAFs.

### 4.2. CAFs and Inflammation

Extensive studies of pancreatic inflammation show that CAFs express several paracrine factors and their receptors, which modulate inflammatory and fibrotic processes. Inflammation and fibroblast activity are closely linked. In pancreatitis, for instance, damage in pancreatic tissues proceeds a succession of events that includes interstitial edema, parenchymal cell necrosis, trypsin activation, inflammatory cell infiltration, and lastly, the activation and proliferation of PSCs [10]. The activated PSCs are often found in areas rich in cytokines, growth factors, and reactive oxygen species, such as near necrotic tissues [10]. The excessive ECM deposition and remodeling that follows PSCs activation is likely a late step in the tissue repair process, similar to that seen in wound healing.

CAFs actively contribute to inflammation by producing several cytokines and chemokines. Besides PDGF and TGF-β, which are well recognized in their fibrogenic roles, CAFs secrete several factors, including IL-1β, IL-4, IL-6, IL-13, CXCL8, vascular endothelial growth factor (VEGF), and many others [2,10,12]. These factors contribute to cancer progression by providing means for inflammation, immunosuppression, tumor cell proliferation, angiogenesis, and chemotherapy resistance [2,12].

As we discussed in wound healing, activated fibroblasts only proceed to ECM deposition after the cessation of inflammation, which may suggest that inflammation acts as a checkpoint that regulates fibroblast differentiation into myofibroblasts. This is also similar to the activated PSCs during pancreatitis [10]. It is not clear though if a secretory (or inflammatory) phenotype is present during these processes.

### 4.3. The Context of Secretory CAFs

Ohlund et al. described the secretory (inflammatory) CAFs as they develop when they are not adjacent to tumor cells [38]. This may implicate far-reaching paracrine factors such as chemokines. There is not enough evidence though to conclude the exact mechanism by which secretory CAFs develop [38]. We will next discuss a few reports that have indicated secretory functions in CAFs and we will aim to identify the common denominator that can explain the development of the secretory CAFs.

Chan et al. treated CAFs in breast cancer and PDAC with the maximum-tolerated dose of chemotherapy [29]. The treatment caused the CAFs to undergo senescence, activate transcription factors, such as the nuclear factor kappa-light-chain-enhancer of activated B cells (NF-κB) and the signal transducer and activator of transcription 1 (STAT1), and highly express a group of chemokines that signal through the chemokine receptor CXCR1/2 axis [29]. These secreted factors enhanced tumor cell proliferation and stemness, angiogenesis, recruitment of myeloid-derived suppressor cells (MDSCs), and rendered larger tumors [29]. Senescence often happens in response to accumulation of somatic mutations, oxidative stress, telomere dysfunction and shortening, loss of immune surveillance, and chronic inflammation in response to inflammatory mediators such as IL-1, IL-6, and CXCL8 [66,67,68]. Senescence in CAFs has been reported on multiple occasions to impact tumorigenicity and tumor cell behaviors [69,70,71]. Senescent fibroblasts promoted proliferation and altered epithelial cell differentiation in breast cancer [72]. Bavic et al. showed that senescent CAFs in prostate cancer promote proliferation of tumor cells through paracrine signaling [73]. Wang et al. reported that senescent CAFs upregulate CXCL8 and enhance tumor cell migration and invasion [74]. Lastly, the induction of CAF senescence generates a non-fibrogenic myofibroblast phenotype with lower ECM synthesis [75]. Although it seems convincing, senescence cannot account for it all. According to Ohlund et al., tumor cells also promoted the proliferation of the secretory phenotype indicating that they are not senescent [38].

Nonetheless, one common feature of senescent cells is that they activate transcription factors, such as NF-κB and STAT1, that upregulate several paracrine factors including IL-1β, IL-6, CXCL8, and VEGF [29,76,77,78]. NF-κB, in particular, has been under a lot of scrutiny in inflammatory diseases and cancers [76,79]. NF-κB is highly associated with inflammation. Inflammation triggers NF-κB activation, which in turn further amplifies inflammation. Acute inflammation is triggered by several factors, including cytokines, chemokines, pathogen-associated molecular patterns, and damage-associated molecular patterns (DAMPs) [80]. A recent report by Su et al. indicated that complement components could signal through the G protein-coupled receptor 77 (GPR77) on CAFs in breast and lung cancers to activate NF-κB, which results in the upregulation of IL-6 and CXCL8, that promote stemness in tumor cells and cause chemotherapy resistance [30]. Although the authors could not observe a downregulation in the αSMA or the ECM production, they identified this secretory subset of CAFs using GPR77 and cluster of differentiation 10 (CD10) as surface markers [30]. CD10 is a small metalloprotease that is also known as common acute lymphoblastic leukemia antigen (CALLA) and used as a prognostic marker [81]. CD10^+^ stromal cells have been identified in several cancers, including colorectal cancer [82], breast cancer [83], gastric cancer [84], and PDAC [85]. In PDAC, CD10^+^ CAFs promoted tumor cell growth and invasion and were associated with reduced survival and nodal metastasis [85]. It is not clear at this point if CD10 is uniformly expressed in all secretory CAFs or not.

CXCL8, also known as IL-8, is a chemokine that signals via the CXCR1/2 axis along with a group of angiogenic chemokines including CXCL1-3 and CXCL5-8. These chemokines are often referred to as Glu-Leu-Arg (ELR)^+^ chemokines, referencing the conserved amino acid motif. ELR^+^ chemokines are known chemoattractants of myeloid cells such as neutrophils and MDSCs [86,87]. The CXCR2 axis is often considered as pro-tumorigenic in many cancers [88,89]. In PDAC, the CXCR2 axis is involved in MDSCs recruitment, angiogenesis, tumor cell proliferation, and migration. Upregulation of the CXCR2-axis in PDAC is associated with tumor-supporting inflammation, immunosuppression, angiogenesis, and tumor growth. This has made CXCR2 a hot target for PDAC therapy [87,90,91]. The CXCR2 axis’s adverse role in PDAC was more apparently in line with the oncogenic Kras mutation [91,92]. Purohit et al. generated a syngeneic *Cxcr2^−/−^* model using PDAC cells with oncogenic Kras. This stromal ablation of CXCR2 inhibited tumor growth, reduced immunosuppression by lowering infiltration of MDSCs, and reduced angiogenesis, but also increased the fibrotic reaction in the primary tumor and increased metastasis. The increased fibrosis in response to CXCR2 inhibition suggests that the CXCR2 axis may play a role in regulating CAFs [92]. It is known that CAFs secrete CXCL8 [10], but little is known about the role of CXCR2 in CAFs. Few reports have linked CXCR2 to the stromal compartment in PDAC. Inhibiting CXCR2 in the genetically engineered PDAC mouse model that carried the oncogenic Kras mutation the and TGF-β receptor knockout disrupted the tumor-stromal interactions and improved mice survival [93]. Steele et al. used a mouse model with oncogenic Kras and p53 mutations and concluded that CXCR2 is abundant in the stromal regions and that inhibiting CXCR2 suppresses metastasis and improves survival by reducing MDSCs infiltration, although the author did not elaborate on the effect of CXCR2 inhibition on CAFs [94]. Finally, sustained CXCR2 signaling in CAFs was shown to reduce αSMA expression and increase the secretion of several pro-tumorigenic paracrine factors including ELR^+^ chemokines [95,96]. The proposed role of CXCR2 axis in CAFs goes along with what Ohlund et al. reported, that the secretory CAFs develop at a distance from tumor cells [38]. ELR^+^, as well as other chemokines, are considered far-reaching compared to other cytokines such as TGF-β. Chemokines make gradients to recruit target cells from distant locations, such as the circulation or the bone marrow, whereas the effect of cytokines is often local. CXCR2 axis is also known to activate NF-κB, and the sustained CXCR2 signaling was even implicated in the induction of senescence [67,77,97,98,99]. Overall, secretory CAFs develop in the context of inflammation. Inflammatory mediators such as ELR^+^ chemokines and DAMPs activate inflammatory pathways including NF-κB and STAT1 and render CAFs secretory.

### 4.4. The Role Secretory CAFs in PDAC

Secretory CAFs produce several paracrine factors, including interleukins, chemokines and growth factors such as VEGF [2,10]. The factors with more consensus include IL-6 and CXCL8, among other ELR^+^ chemokines. The secreted factors produced by CAFs have been implicated in multiple pro-tumorigenic events, including tumor cells proliferation and migration, stemness, immunosuppression, chemotherapy resistance, and invasion, however, some of these events lack consensus. The roles of IL-6 and CXCL8 in cancer are often associated with increased tumor cells proliferation, recruitment of MDSCs, angiogenesis, tumor cells stemness [29,30,91,92,94,100] (Figure 1). Thus, we expect to find that tumors with an abundance of secretory CAFs will be bigger in volume due to proliferation cues and vascularity, immunosuppressive due to MDSC infiltration, and resistant to chemotherapy with enhanced undifferentiated histology as a result of stemness (Figure 2). These characteristics are similar to those observed with αSMA depletion and HH inhibition [6,7], which is likely due to the abundance of secretory CAFs. On the other hand, the abundance of myofibroblasts will likely result in increased stiffness, hypoxia, induction of EMT, infiltration of macrophages, and metastasis. Such characteristics observed with stromal CXCR2 deletion.

## 5. Conclusions

PDAC remains one of the most challenging human malignancies due to its late detection and low effectiveness of current therapies. The characteristic complex tumor microenvironment and the dense desmoplastic reaction in PDAC contribute to tumorigenicity and tumor progression. CAFs represent a major component in the PDAC tumor microenvironment and contribute to tumor progression and dissemination. In this review, we discussed CAFs’ functional heterogeneity based on information available in the literature. We gathered that there are at least two functional entities within the CAF population. Myofibroblasts are the typical CAFs that have been described in the literature as synonymous. Myofibroblasts are characterized by an enhanced ECM production and the expression of αSMA. The abundance of myofibroblasts contributes to tumor stiffness, hypoxia and avascularization, physical resistance to chemotherapy, EMT, and invasion. In contrast, secretory CAFs develop in response to inflammation that results in the activation of transcription factors, including NF-κB and STAT1, which propagate inflammation by secreting additional mediators, such as IL-6 and CXCL8. Tumors abundant in secretory CAFs are expected to be more aggressive due to increased proliferation and vascularization, immunosuppressive due to increased recruitment of MDSCs, and resistant to chemotherapy as a result of the induction of stemness.

Nonetheless, several questions remain unanswered. It is unclear whether the origin of CAFs impacts their functional polarization, or if these two events are mutually exclusive. Raz et al. demonstrated that CAFs derived from bone marrow in breast cancer have low expression of PDGFRα and are functionally distinct from PDGFRα-expressing CAFs, which suggests that functional heterogeneity may, to some extent, be determined by the origin of CAFs [101]. Furthermore, CAFs’ functional status as myofibroblast or secretory might be dependent on the differentiation state or just a dynamic process in response to milieu changes; whether they are able to switch from one to another remains unknown. Moreover, it is not known if there are additional CAFs subsets present in PDAC other than what we have discussed. In addition, there is a lack of suitable markers that can distinguish the functional subsets of the CAFs population. Targeting CAFs, ECM, or desmoplasia is still currently considered. Nielsen et al. described the key players in cancer–stromal interaction in PDAC and summarized the important clinical trials that target components of PDAC stroma [36]. Although targeting CAFs and desmoplasia may still be an attractive option for treating PDAC, more studies that dive into the characterization of CAFs are warranted at this time.
